# Abdominal B-Cell Lymphoma Mimicking Ovarian Cancer

**DOI:** 10.3390/diagnostics14212449

**Published:** 2024-10-31

**Authors:** Dennis Jung, Lina Judit Schiestl, Simin Schadmand-Fischer, Arno Schad, Annette Hasenburg, Roxana Schwab

**Affiliations:** 1 Department of Obstetrics and Gynecology, University Medical Center of Johannes Gutenberg University Mainz, 55131 Mainz, Germany; 2 Department of Radiology, University Medical Center of Johannes Gutenberg University Mainz, 55131 Mainz, Germany; 3 Institute of Pathology, University Medical Center of Johannes Gutenberg University Mainz, 55131 Mainz, Germany

**Keywords:** B-cell lymphoma, ovarian cancer, intraoperative images: computed tomography

## Abstract

A 54-year-old patient presented in our clinic with pressure in the upper abdomen, dyspnea and abdominal distension. The clinical examination showed pleural effusion, ascites and an enlarged axillary lymph node on the right side. In gynecological sonography ascites, an ovarian cyst and peritoneal carcinosis in the pouch of Douglas were detected, which were potentially indicative of ovarian cancer. A staging laparoscopy was performed to confirm the diagnosis of ovarian cancer and to evaluate operability. Intraoperatively white milky ascites, white-yellow marbling of the liver and white stipple bedding on the diaphragm and liver were detected. The ovaries and the fallopian tubes were tumorously enlarged. Biopsies were taken from the right fimbrial funnel, the liver around the falciform ligament and the diaphragm. Histology of all abdominal biopsies and the axillary lymph node revealed high lymphatic infiltration matching a stage III B-cell-lymphoma. The patient was transferred to the hemato-oncological department for further therapy. Six cycles of cytostatic therapy with R-CHOP (rituximab, cyclophosphamide, hydroxydaunorubicin, vincristine sulfate, prednisone) were initiated. The patient is doing well and in stable disease 6 months after completion of cytotoxic therapy. This case report presents a rare case of manifestation of an extra nodal B-cell-lymphoma with abdominal presentation that mimicked ovarian cancer.

A 54-year-old patient presented in our clinic with pressure in the upper abdomen, dyspnea and abdominal distension. Patient’s anamnesis and family history were blank as well as screening for familial breast and ovarian cancer. The clinical examination showed pleural effusion, ascites and an enlarged axillary lymph node on the right side. In gynecological sonography and preoperative computed tomography ascites, an ovarian cyst and peritoneal carcinosis in the pouch of Douglas were detected, which were potentially indicative of ovarian cancer (Figure 1 and Figure 2). Laboratory results and blood count were normal except for tumor marker CA-125, which was increased at 531 U/mL. A staging laparoscopy was performed to confirm the diagnosis of ovarian cancer and to evaluate operability. Intraoperatively white milky ascites, white-yellow marbling of the liver and white stipple bedding on the diaphragm and liver were detected. The ovaries and the fallopian tubes were tumorously enlarged (Figure 3). Biopsies were taken from the right fimbrial funnel, the liver around the falciform ligament and the diaphragm. Histology of all abdominal biopsies and the axillary lymph node revealed high lymphatic infiltration matching a stage III B-cell-lymphoma (Figure 4). The postoperative course was uneventful, and the patient was transferred to the hemato-oncological department for further therapy. Six cycles of cytostatic therapy with R-CHOP (rituximab, cyclophosphamide, hydroxydaunorubicin, vincristine sulfate, prednisone) were initiated. The patient is doing well and in stable disease 6 months after completion of cytotoxic therapy. This case report presents a rare case of manifestation of an extra nodal B-cell-lymphoma with abdominal presentation that mimicked ovarian cancer. Primary Ovarian Non-Hodgkin’s Lymphoma is a rare condition and amounts to 0.5% of all Non-Hodgkin lymphoma [1]. Different cases have already been reported. In all but one of these, explorative laparotomy was carried out in order to confirm diagnosis and then aborted after histologic diagnosis. One case was diagnosed after biopsy of the ovarian mass [2,3]. In another case series, all patients underwent total abdominal hysterectomy and bilateral salpingo-oophorectomy [1]. In our case, we first performed a laparoscopy to confirm diagnosis. Staging laparoscopy and biopsy were crucial steps to obtain the right diagnosis and initiate the right therapy. Laparoscopy was also less harmful than laparotomy and subsequent chemotherapy could start sooner. Interdisciplinary cooperation was crucial for a fast and adequate induction of the appropriate therapy.

## Figures and Tables

**Figure 1:**
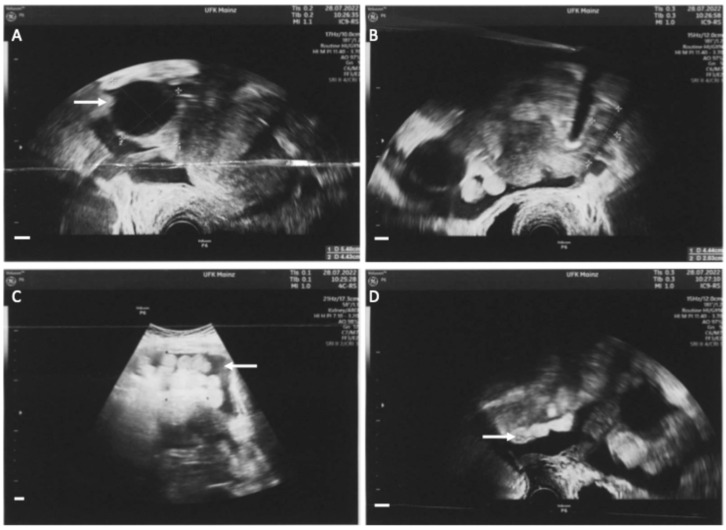
Sonographic assessment at first presentation in our clinic. (**A**): Enlarged right ovary with an unilocular tumor of 5.4 × 4.4 cm with low echogenicity and a papillary structure (white arrow). (**B**): Left ovary without pathologic findings. (**C**): Ascites (white arrow). (**D**): Peritoneal carcinosis (white arrow). The combination of these findings (ovarian tumor, ascites and peritoneal carcinosis) are potentially indicative of ovarian cancer. All white bars in the bottom left-hand corner indicate 1 cm.

**Figure 2:**
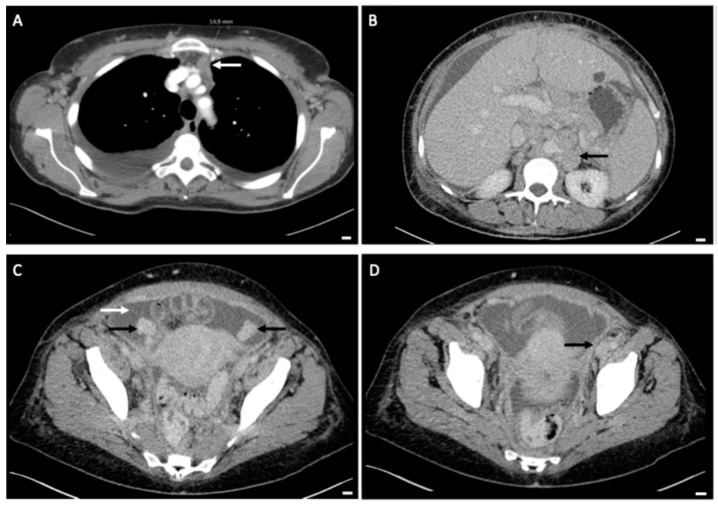
Preoperative CT-imaging of thorax and abdomen. (**A**): Up to 15 mm pathologically enlarged retrosternal lymph node (white arrow). (**B**): Bulky lymph nodes paraaortal left with single lymph nodes enlarged up to 2.8 cm (black arrow). (**C**): Solid, up to 3 cm enlarged ovaries on both sides (black arrows), ascites (white arrow). (**D**): Thickened peritoneum in the small pelvis, potentially indicative of peritoneal tumor metastases (black arrow). All white bars in the bottom right-hand corner indicate 1 cm.

**Figure 3:**
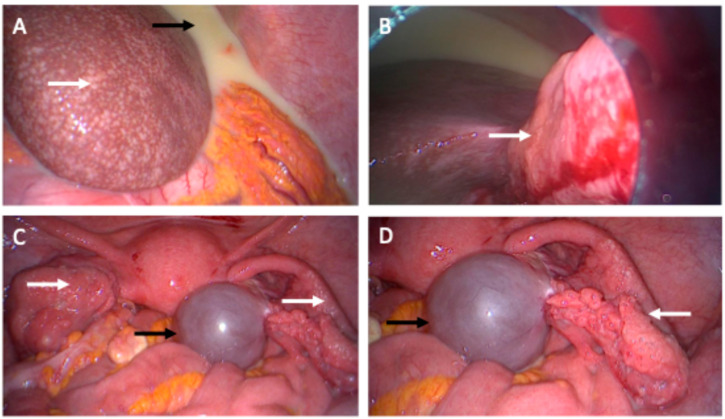
Intraoperative images of staging laparoscopy. A laparoscopy was performed to confirm diagnosis after biopsy. Biopsies were taken from the diaphragm, the liver around the falciform ligament and from the right fimbrial funnel. All biopsies revealed the diagnosis of a B-cell lymphoma. The German guideline on ovarian cancer does not recommend laparoscopy [4], however the American guideline NCCN states that laparoscopy may be appropriate to obtain biopsy material and to confirm the diagnosis [5]. (**A**): White-yellow marbling of the liver (white arrow) and white milky ascites (black arrow). (**B**): White stipple bedding on the diaphragm (white arrow). (**C**): Overview of the pelvis. Uterus without pathological findings; the fallopian tubes are enlarged (white arrow), paraovarian-cyst (black arrow). (**D**): Focused image of the right enlarged fallopian tube (white arrow) and the paraovarian-cyst of the right ovary (black arrow).

**Figure 4:**
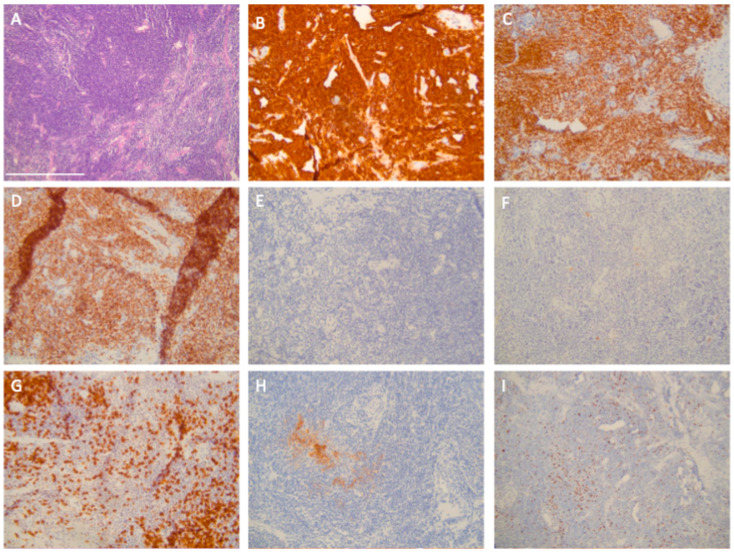
Pathological and immunohistochemical microscopic images display the typical immune phenotype of the lymphoma; at magnification 20×, the white bar in the bottom left-hand corner in (**A**) indicates 250 μm for all images. (**A**): hematoxylin eosin overview. Lymphoma is positive for CD20 (**B**), PAX5 (**C**) and bcl2 (**D**). Immunohistochemical staining is negative for bcl6 (**E**) and cyclin D1 (**F**). (**G**): Interspersed CD3-positive T-cells. (**H**): CD23-staining with focally destroyed germinal centers. (**I**): Moderate staining of Ki-67.

## Data Availability

Not applicable.

## Abbreviation

The following abbreviation is used in this manuscript:

R-CHOP	rituximab, cyclophosphamide, hydroxydaunorubicin, vincristine sulfate, prednisone
